# Early Childhood Caries: Epidemiology, Aetiology, and Prevention

**DOI:** 10.1155/2018/1415873

**Published:** 2018-05-22

**Authors:** F. Meyer, J. Enax

**Affiliations:** Research Department, Dr. Kurt Wolff GmbH & Co. KG, Bielefeld, Germany

## Abstract

Early childhood caries (ECC) is one of the most prevalent diseases in children worldwide. ECC is driven by a dysbiotic state of oral microorganisms mainly caused by a sugar-rich diet. Additionally, poor oral hygiene or insufficient dental plaque removal leads to the rapid progression of ECC. ECC leads not only to dental destruction and pain with children, but also affects the quality of life of the caregivers. Children with extensive ECC are at high risk to develop caries with the permanent dentition or will have other problems with speaking and/or eating. To prevent ECC, several strategies should be taken into account. Children should brush their teeth with toothpastes containing gentle ingredients, such as mild surfactants and agents showing antiadherent properties regarding oral microorganisms. Parents/caregivers have to help their children with brushing the teeth. Furthermore, remineralizing and nontoxic agents should be included into the toothpaste formulation. Two promising biomimetic agents for children's oral care are amorphous calcium phosphate [Ca_*x*_(PO_4_)_*y*_*n* H_2_O] and hydroxyapatite [Ca_5_(PO_4_)_3_(OH)].

## 1. Introduction

Early childhood caries (ECC) is still one of the most prevalent diseases in children worldwide. ECC does not only affect children's oral health, but also the general health of children [1, 2]. Not only oral pain, orthodontic problems, and enamel defects, but also problems with eating and speaking can occur as well as an increased risk for caries development in the permanent dentition [3]. Premature loss of primary dentition often leads to orthodontic problems in adult life [4]. Not only children are affected, but also parents will be influenced by this disease being the responsible caregivers [3, 4]. For example, dental problems were shown to be the main reasons for hospitalisation of children in Australia in 2015 [5]. Thus, ECC leads not only to temporary pain, but more importantly has major effects on the quality of life of the family/caregivers including financial and health implications [6, 7]. The aim of this review article is to present the state of the art of the epidemiology, aetiology, characteristics of primary dentition, risk factors, general recommendations, and strategies for prevention of ECC.

## 2. Background and Epidemiology

As stated before, ECC is still one of the most abundant diseases worldwide. The incidence of ECC among children with deciduous teeth is 1.76 billion (95% CI: 1.26 billion; 2.39 billion) [8]. Interestingly, ECC is not limited to children with a low socioeconomic status (SES) [9, 10]. Recent data, for example, from Australia show a prevalence of more than 50% of 6-year-old children with caries on deciduous teeth [5]. Data from different parts of the world show up to 89.2% of children with ECC in Qatar and 36% in Greece [11, 12]. About the same prevalence (ca. 40%) has been reported in the USA among 2–11 year old children [13]. A recently published study from Germany shows even 10% (up to 26% with initial lesions) of 3-year-old children with ECC and an increase up to about 50% in 6-/7-year-old children [14]. Even though the dmft-index (decayed missing filled teeth) has decreased over time in general [10, 14], the prevalence has not decreased [14]. However, a study from Germany was also able to show different trajectories and an increase of dmft-values when looking at a smaller scale on a regional level [10]. While most of the districts in a midsized German city showed a decrease of dmft, the dmft increased in other districts over time [10]. Milsom et al. described that children with an already existing caries lesion have a 5–6 times higher incidence of developing new caries lesions compared to previously caries-free children [15]. Sleeping problems and insufficient sleep can also be identified as risk factor for ECC, as sleeping problems lead to a more frequent use of night-time bottle use with sugar-sweetened beverages [16–18]. As the role of parents is still unclear with respect to their children developing ECC, several studies have focussed on different associations [19]. Sociocultural and socioeconomic backgrounds of the parents can be found as risk factors for ECC, but parental stress does not show a significant increase in ECC with the children [10, 19, 20]. Not only children, but also their parents should be motivated to take care of the primary dentition to prevent ECC and consequently further caries development in the secondary dentition [21].

## 3. Aetiology of ECC

Dental caries develops when the dental plaque, a polymicrobial biofilm, is not removed regularly and the diet consists of mainly monosaccharides. Monosaccharides can be metabolized by many of the oral bacteria leading to an increased production of acids which are able to demineralize the enamel [22, 23]. Dental plaque is built on top of the pellicle starting directly after mechanical removal of the biofilm [24, 25]. More than 700 bacterial species/taxa are known in the oral flora [26]. Because the oral habitat consists of many different ecological niches, the relatively high number of different species/taxa can be explained [27]. Oral microorganisms are able to interact with each other and mainly communicate using so-called “quorum sensing” (QS) [28]. Nowadays, it is well known that not only bacteria, but also fungi, such as *Candida albicans* and the interkingdom interactions, can enhance the progression of caries [29, 30]. However, microorganisms grown in polyspecies biofilms are able to produce exopolysaccharides (EPS), also known as extracellular polymeric substances [31, 32]. With the help of the EPS, microorganisms are able to resist antimicrobials that are recently used in toothpastes [32]. Consequently, biofilm formation is not interrupted and together with the absorbed saccharides from the diet leads to a cariogenic dental plaque [33]. The dental plaque on clinically sound enamel of children consists mainly of streptococci and actinomycetes [34]. With a low-sugar diet, these microorganisms are living as commensals in a homeostatic environment controlling each other [35]. As soon as sugars, especially sugary food and beverages, are consumed, the commensal plaque microbiota will absorb these saccharides and metabolize them into acids, mainly lactic acid [36]. This acid production leads to a pH shift from around 7 (neutral) to a pH < 5.5 (acidic) [37]. Acid-tolerant bacteria, mainly mutans streptococci, are able to survive these acidic environments [36]. When oral hygiene habits and nutritional habits do not change, a reduction of highly cariogenic microorganisms (mutans streptococci, *Candida* spp., and lactobacilli) cannot be achieved [38]. Peterson et al. used next-generation sequencing (NGS) to identify the microbial composition of the dental plaque. They show only slight differences between the biofilms collected from children with and without caries [39]: *Streptococcus mitis* and *Streptococcus sanguinis* were found in both groups. *Streptococcus* was found to be the most abundant genus (>50% of the microorganisms). *Veillonella*, *Granulicatella*, *Fusobacterium*, *Neisseria*, *Campylobacter*, *Gemella*, *Abiotrophia*, *Selenomonas,* and *Capnocytophaga* were also found in abundance between 1 and 10% of the biofilm [39]. Simon-Soro et al. also detected *Lactobacillus*-species, known as acid-resistant bacteria, associated with caries [40, 41]. Even though the studies described above used NGS strategies, this technique is rapidly developing and recent studies are able to use even more sophisticated models predicting ECC [42]. Teng et al. used *in vivo* samples from a 3-year cohort study and showed, with the help of mathematical modelling, that *S. mutans* were not the main trigger for caries, but identified *Veillonella* spp. and *Prevotella* spp. instead [43]. *Veillonella atypical*, *V. dispar*, and *V. parvula* as well as *Prevotella* spp. were identified as bacteria that are mainly responsible for the development of ECC [43].

In conclusion, ECC develops as soon as the dental plaque is not removed adequately and a sugary diet, especially sweetened food and beverages, is consumed. This leads to a changing metabolism with the dental plaque microbiota producing mainly lactic acids that will demineralize the enamel. *Prevotella* spp. and *Veillonella* spp. were shown to be microbial risk factors, while together with fungi, bacteria can trigger acid metabolisms and virulence of the microorganisms [29,30,41–43].

## 4. Characteristics of Deciduous Enamel and Enamel of Permanent Teeth

Enamel is the hardest tissue in the human body. It mainly consists of hydroxyapatite (97%) (HAP), Ca_5_(PO_4_)_3_(OH), which is a calcium phosphate mineral [44–49]. Enamel is highly mineralized and has extraordinary mechanical properties [44, 45, 47, 50, 51]. The interior of a tooth consists of dentin (about 70% HAP and 20% proteins mainly collagen and 10% water), produced by odontoblasts, and the enamel, that is built by ameloblasts. Ameloblasts are restricted to produce enamel one time: ameloblasts produce several proteins and attract calcium and phosphate ions to crystallize these [52, 53]. The enamel of deciduous teeth is built within a significantly shorter period (24 months) than permanent teeth (up to 16 years) [52]. The consequence of the shorter time for enamel development is the formation of a very thin enamel (half the thickness than that of the permanent teeth) and a less organized microstructure [54, 55]. As consequence, acids are able to demineralize deciduous enamel faster than permanent enamel [56–58].

## 5. Risk Factors

ECC is known to be a multifactorial disease. Sugary food and beverages can lead to a dysbiotic state of the microbial composition causing caries. As ECC is also known as “baby bottle caries,” feeding practices are noticed as main risk factor developing ECC [9, 59, 60]. Here, the upper incisors and molars are affected at first, followed by the molars of the lower jaw and finally the lower jaw incisors [61]. Children sleeping with bottles filled with sweetened tea or milk containing several cariogenic sugars are at high risk for developing ECC. As a consequence of drinking during night time, without clearance of sugars, the oral bacteria will produce lactic acid rapidly, demineralizing the enamel [9, 62]. Nowadays, not only baby bottles, but also several other sweetened juices consumed throughout the day or even at night will enhance the risk to develop caries. ECC is a disease affecting both low-SES families and high-SES families [9, 10]. However, unemployment and migration background can be found as risk factors for spatial disparities in ECC [10]. Other important factors that increase the risk to develop ECC are irregular toothbrushing (mechanical plaque removal) and/or toothbrushing without supervision by any caregivers [63]. Therefore, supervised thorough tooth brushing twice a day should be applied [64].

## 6. General Recommendations

The primary dentition usually erupts 6 to 8 months after birth [65]. As the oral cavity is highly sensitive, soft touches of the oral mucosa and gingiva should be performed in the early infant life to get the infants used to tooth brushing. Tooth brushing of at least two to three minutes should be performed two times a day by the caregivers as soon as the first tooth erupts [65]. Most dentists recommend to use a “pea-size” amount of a fluoride toothpaste for children, which contain usually not more than 500 ppm fluoride [66]. Additionally, fluoride gels could be used [67]. However, adverse effects like fluorosis need to include assessment of potential adverse effects [67].

## 7. Biomimetic Concepts and Tooth Brushing to Prevent ECC

It is well known that fluorides and especially fluoridated toothpastes may have a beneficial effect inhibiting caries progression [68]. However, an average caries reduction of 23% compared to a placebo can only be detected using toothpastes containing a minimum of 1000 ppm (0.1%) fluoride. In Europe as well as other parts of the world, toothpastes for children should contain a maximum of 500 ppm (0.05%) fluoride [66, 69]. Toothpastes with more than 1000 ppm fluoride to 1500 ppm fluoride have to be labelled in the EU as “Children of 6 years and younger: Use a pea sized amount for supervised brushing to minimize swallowing. In the case of fluoride intake from other sources consult a dentist or doctor” [70]. Other countries have similar restrictions and warnings. The reason is an enhanced risk for dental fluorosis and skeletal fluorosis due to the accumulation of fluoride from different sources and swallowing the fluoridated toothpaste [66]. Additionally, it is discussed whether fluorides interact with ameloblasts and have negative impact on the enamel formation [53, 71]. Fluorides are mainly functioning due to topical application by enhancing remineralization with calcium and phosphate ions derived from saliva [72]. Consequently, intake of fluoride tablets and fluoridated salts are discussed whether to be effective in caries protection or not [66]. In the past, it was assumed that fluorides lead to the formation of fluoroapatite [Ca_5_(PO_4_)_3_F]. This mechanism was thought to make teeth more resistant to acids and protect the enamel. However, only small amounts of fluorapatite can be detected [50,73–75]. Interestingly, fluorotic teeth with higher concentration of fluoride are even less resistant to acids than sound enamel [75–77].

Alternatives to prevent caries and especially ECC in children need to be based on biomimetic strategies. Several products based on different calcium phosphates are already on the market and well studied [78]. Besides others, hydroxyapatite (HAP) [Ca_5_(PO_4_)_3_(OH)] and amorphous calcium phosphates [Ca_*x*_(PO_4_)_*y*_·*n* H_2_O] stabilized by casein proteins (CPP-ACP) show the most promising results. HAP was identified to be very effective in preventing ECC within a cohort of Japanese children following a 3-year study which showed a reduction of new caries lesions of up to 56% [69, 79]. A recently published randomized, double-blind clinical study shows that microcrystalline HAP is not inferior to fluorides in clinical caries prevention [80]. Besides remineralizing properties that are equal to sodium fluoride [81], HAP-microclusters were shown to reduce dental plaque formation *in situ* and *in vivo* [82–86]. Lelli et al. were able to observe a protective layer on the top of enamel after using HAP-toothpaste *in vivo* [87]. Similar results can be found when using CPP-ACP. This calcium phosphate is also able to remineralize initial enamel lesions equivalent to fluorides [88]. Different studies showed that early lesions can also be remineralized and regressed using CPP-ACP [89] and regarding remineralization identified CPP-ACP to even be superior to a high fluoride containing product (5000 ppm fluoride) [90]. However, others studies have shown contrary results [91, 92].

Besides remineralizing ingredients, toothpastes should have biofilm controlling properties that do not affect children's health [93] (Table 1).

The use of an appropriate toothpaste should be accompanied by twice daily supervised/supported tooth brushing as well as regular visits to the dentist (at least once a year) [96]. With respect to the motor abilities of very young children, brushing should be carried out using electric brushes or manual toothbrushes especially made for children under parental supervision [97]. Even though there are several brushing techniques known, for younger children, the horizontal brushing technique is recommended combined with a 3-minute brushing period [98]. Paediatricians should also check both the oral health and fluoride anamnese of the children while also asking the parents about the child's oral hygiene. Toothbrushes should be replaced every 3 months or when the bristles become frayed with use [99].

## 8. Conclusion

In additionally to a low sugary diet, children should brush their teeth twice a day under parental supervision and be supported with brushing. The caregivers should especially support very young children (under the age of 3) continuously. Toothpastes should mainly comprise promising remineralizing agents for children's oral care such as calcium phosphates like CPP-ACP or HAP.

## Figures and Tables

**Table 1:** Main ingredients for children's toothpastes (up to 6 years).

Ingredient	Function/comments	References
Silica- or calcium carbonate-based abrasives with low RDA value (RDA: radioactive dentin abrasion)	Plaque removal, gentle cleaning of deciduous teeth	[94]
Hydroxyapatite, ACP-CPP, fluorides in low concentrations	Remineralization	[45, 88]
Hydroxyapatite	Reduction of bacterial adhesion to enamel surfaces due to antiadhesive properties	[82, 84]
Surfactants	Foaming action, due to irritant properties, children's toothpaste should not contain any sodium lauryl sulfate (SLS)	[69]
Preservatives/antimicrobial agents	Due to risk of swallowing, children's toothpaste should not contain any potent antimicrobial agents such as chlorhexidine or triclosan, but mild preservatives (e.g., alkanediols or xylitol as antimicrobial agent)	[69, 95]
